# Supplementary material for: The Benefits and Hazards of Intravitreal Mesenchymal Stem Cell (MSC) Based-Therapies in the Experimental Ischemic Optic Neuropathy
Source: Int J Mol Sci. 2021 Feb 20;22(4):2117. doi: 10.3390/ijms22042117 (PMC7924624; doi:10.3390/ijms22042117)
Supplement: Supplementary file 1 [file ijms-22-02117-s001.pdf]

HLA-DR & HLA-ABC expression before WJ-MSCs transplantation based on flowcytometry

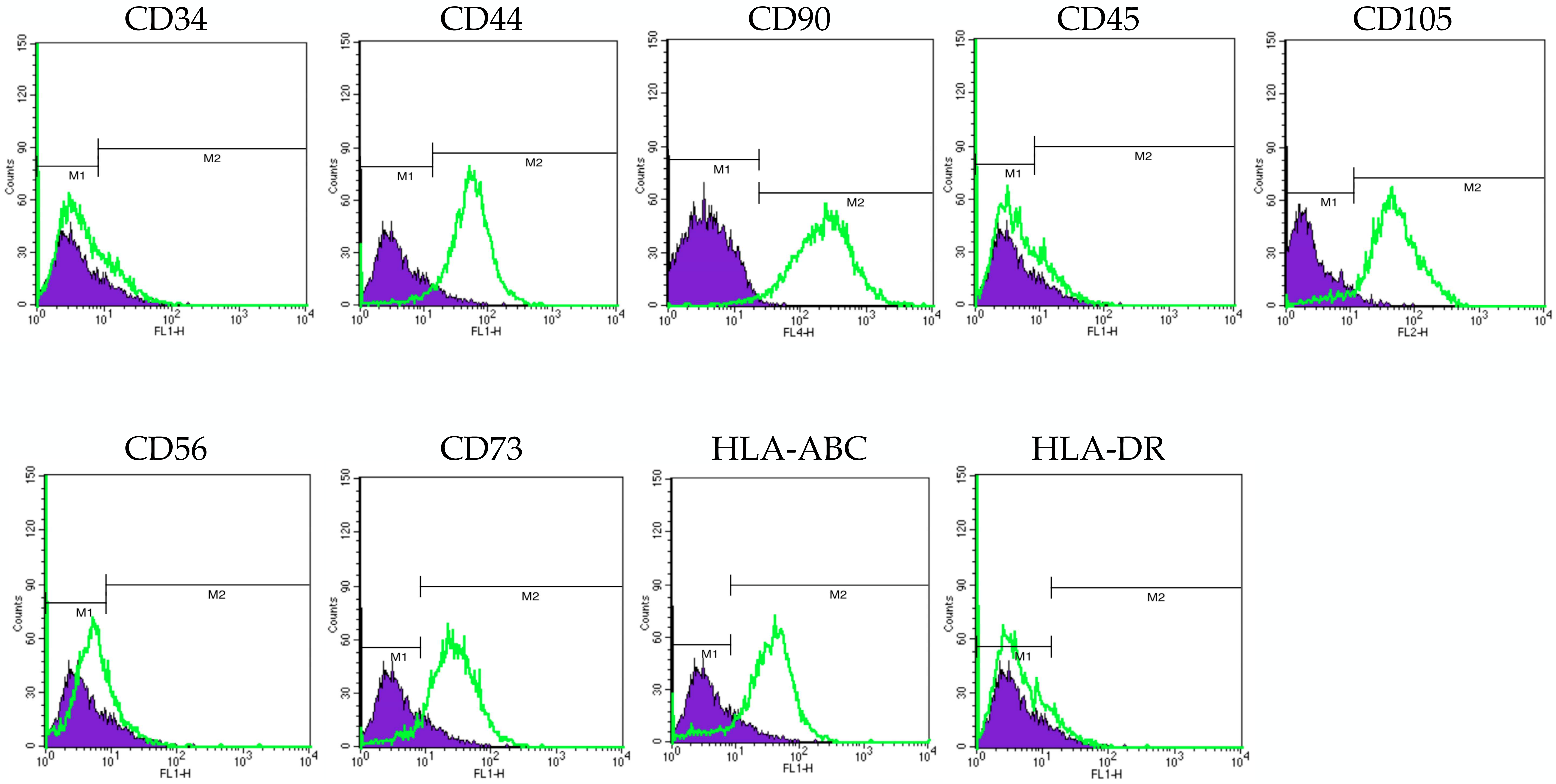

Marker	Status
CD34	neg
CD44	pos
CD90	pos
CD45	neg
CD105	pos
CD56	neg
CD73	pos
HLA-ABC	pos; 92.43%
HLA-DR	neg; 9.51%
